# Aqueous extracts from peppermint, sage and lemon balm leaves display potent anti-HIV-1 activity by increasing the virion density

**DOI:** 10.1186/1742-4690-5-27

**Published:** 2008-03-20

**Authors:** Silvia Geuenich, Christine Goffinet, Stephanie Venzke, Silke Nolkemper, Ingo Baumann, Peter Plinkert, Jürgen Reichling, Oliver T Keppler

**Affiliations:** 1 Department of Virology, University of Heidelberg, Heidelberg, Germany; 2 Department of Pharmacy and Molecular Biotechnology, University of Heidelberg, Heidelberg, Germany; 3 Department of Otolaryngology, Head and Neck Surgery, University of Heidelberg, Heidelberg, Germany

## Abstract

**Background:**

Aqueous extracts from leaves of well known species of the Lamiaceae family were examined for their potency to inhibit infection by human immunodeficiency virus type 1 (HIV-1).

**Results:**

Extracts from lemon balm (*Melissa officinalis *L.), peppermint (*Mentha *× *piperita *L.), and sage (*Salvia officinalis *L.) exhibited a high and concentration-dependent activity against the infection of HIV-1 in T-cell lines, primary macrophages, and in *ex vivo *tonsil histocultures with 50% inhibitory concentrations as low as 0.004%. The aqueous Lamiaceae extracts did not or only at very high concentrations interfere with cell viability. Mechanistically, extract exposure of free virions potently and rapidly inhibited infection, while exposure of surface-bound virions or target cells alone had virtually no antiviral effect. In line with this observation, a virion-fusion assay demonstrated that HIV-1 entry was drastically impaired following treatment of particles with Lamiaceae extracts, and the magnitude of this effect at the early stage of infection correlated with the inhibitory potency on HIV-1 replication. Extracts were active against virions carrying diverse envelopes (X4 and R5 HIV-1, vesicular stomatitis virus, ecotropic murine leukemia virus), but not against a non-enveloped adenovirus. Following exposure to Lamiaceae extracts, the stability of virions as well as virion-associated levels of envelope glycoprotein and processed Gag protein were unaffected, while, surprisingly, sucrose-density equilibrium gradient analyses disclosed a marked increase of virion density.

**Conclusion:**

Aqueous extracts from Lamiaceae can drastically and rapidly reduce the infectivity of HIV-1 virions at non-cytotoxic concentrations. An extract-induced enhancement of the virion's density prior to its surface engagement appears to be the most likely mode of action. By harbouring also a strong activity against herpes simplex virus type 2, these extracts may provide a basis for the development of novel virucidal topical microbicides.

## Background

Advances in HIV pharmacotherapy led to the current highly active antiretroviral therapy (HAART), which has had significant impact on HIV/acquired immunodeficiency syndrome (AIDS) in the developed world, and these drugs have acted to prolong survival and to alleviate suffering. However, the incidence of side effects and HIV drug resistance in patients under HAART is high [1] and HIV/AIDS persists as a major cause of morbidity in Western societies and continues to surge unabated in the developing word. Consequently, there remains an urgent need for more potent and conceptually novel antiviral therapeutics to add to current treatment regimens. Over the past decade, the concept of topical microbicides to prevent transmission of HIV has emerged as an important strategy to control the HIV pandemic [2]. The increased incidence of HIV infection in women aged 15–49 years in resource-poor countries has emphasized the need to develop female-controlled, efficacious and safe microbicides for vaginal application [3,4]. Desirable basic characteristics of topical microbicides include a high *in vitro *activity against a wide range of HIV-1 strains, a broad activity against other sexually-transmitted pathogens, no-to-low cytotoxicity in *in vitro *assays, stability under likely storage conditions, low cost, and good acceptance in the target population [3,4].

Plants of the Lamiaceae family are used in traditional and complementary medicine, in particular in phytotherapy. A virucidal activity of extracts from lemon balm has been reported for herpes simplex virus type 1 (HSV-1) and type 2 (HSV-2) [5-7], and recently been extended to other species of the Lamiaceae family [8]. HSV-2 is the major cause of genital ulcerative disease. Here, we explored the antiviral potency of aqueous extracts prepared from dried leaves from lemon balm, peppermint and sage against HIV-1.

## Results

### Potent anti-HIV activity of aqueous extracts from sage, peppermint, and lemon balm

We systematically investigated the potency of aqueous extracts prepared from dried leaves of well known members of the Lamiaceae family, lemon balm, peppermint, and sage to inhibit HIV-1 infection *in vitro *and *ex vivo*. We employed an experimental set-up that allowed us to study potential effects that aqueous extracts may have on HIV virions as well as on intracellular steps of the viral replication cycle. To this end, stocks from the prototypic X4 HIV-1_NL4-3 _strain were first incubated with concentrations of aqueous extracts ranging from 0.006 to 6% for 1 h at 37°C and subsequently, these suspensions were mixed with an equivalent volume of culture medium and added to the human T-lymphoblastoid cell line Sup-T1. Following overnight exposure, cells were washed, and cultivated for four more days (in the absence of extract). Then, productive HIV-1 infection was assessed by the p24 concentration in culture supernatants.

**Figure 1:**
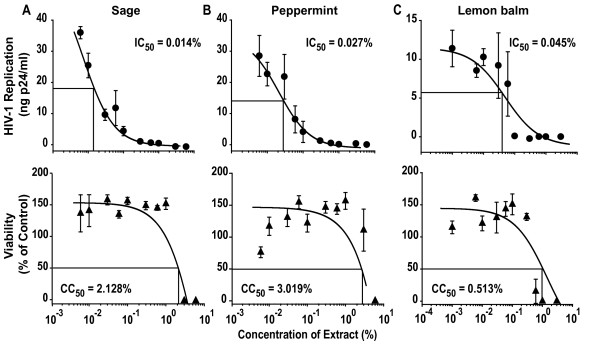
**Aqueous extracts of Lamiaceae exhibit a concentration-dependent anti-HIV-1 activity on Sup-T1 T-cells**. Upper panels (HIV-1 replication): HIV-1_NL4-3 _stocks were exposed to the indicated concentrations of aqueous extracts from (A) sage, (B) peppermint, (C) lemon balm, or solvent (H_2_O) for 1 h at 37°C. Subsequently, Sup-T1 cells were challenged overnight with the virus-extract suspension and washed the following day. HIV-1 replication was monitored by p24 ELISA and values determined from culture supernatants taken at day 5 post infection are shown. Lower panels (viability): In parallel, Sup-T1 cells were exposed to the identical extract concentrations overnight, washed, and analyzed for viability in a standard MTT viability assay. Each experiment was performed in triplicate, and 4–6 independent experiments were conducted (see also Table 1). Given are arithmetic means ± standard deviations (SD) from one experiment. The indicated IC_50 _and CC_50 _values were determined by using Prism software (GraphPad, San Diego, CA).

**Figure 2:**
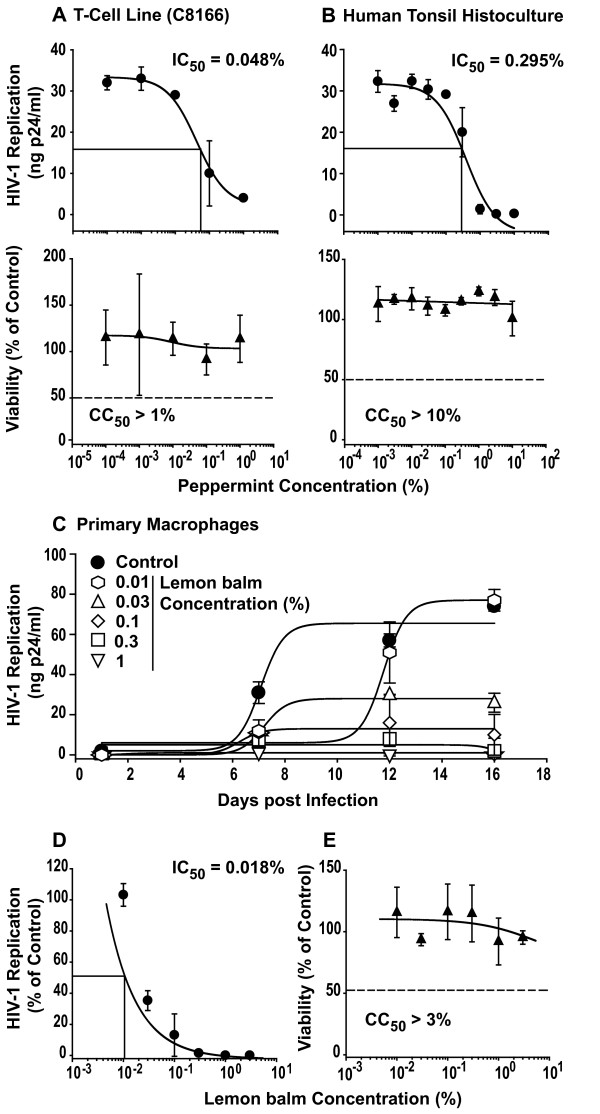
**Aqueous extracts of Lamiaceae potently inhibit HIV-1 replication in C8166 T-cells, in the *ex vivo *human tonsil histoculture, and in monocyte-derived macrophages**. The anti-HIV_NL4-3 _activity and cytotoxicity of aqueous peppermint extract was assessed in (A) C8166 T-cells or (B) human lymphoid aggregate cultures (HLAC) from tonsil in principle as described in the legend to Fig. 1. Each experiment was performed in triplicate, and 2–4 independent experiments were performed (see Table 1). Shown are arithmetic means ± SD from one experiment. (C) HIV-1_YU-2 _replication kinetics in monocyte-derived macrophages under conditions of continuous extract exposure. HIV-1_YU-2 _stocks were exposed to the indicated concentrations of lemon balm extract for 1 h at 37°C. Primary macrophages were challenged overnight with the virus-extract suspension, washed the following day, and then cells were continuously cultured in medium containing the indicated concentrations of extract. HIV-1 replication was monitored at days 1, 7, 12, and 16 post infection by p24 ELISA. (D) Relative levels of HIV-1 replication at the endpoint (day 16 post infection) relative to untreated controls with the IC_50 _indicated. (E) Viability. In parallel, monocyte-derived macrophages were exposed to the identical extract concentrations over the 16 day-period and then analyzed in a standard MTT assay. Shown are arithmetic means ± standard deviations relative to untreated controls (set to 100%) from one donor.

All three aqueous extracts showed a strong and concentration-dependent inhibition of HIV-1 replication relative to solvent-treated controls (Fig. 1, top panels). The 50% inhibitory concentration (IC_50_) was fairly comparable for all extracts ranging from 0.014 to 0.045% in this experiment, and from 0.020 to 0.190% as an average of 4–6 independent infection experiments performed on Sup-T1 cells (Table 1). In parallel, a potential extract-induced cytotoxicity was examined using a standard MTT viability assay [9]. Only at extract concentrations around 1% was cytotoxicity observed in Sup-T1 cells (Fig. 1, bottom panels, Table 1) with a resulting selectivity index (SI), defined as CC_50_/IC_50 _derived from individual experiments, of 71, 73, and 103, for peppermint, sage, and lemon balm (Table 1), respectively. Results similar to Sup-T1 cells were obtained for HIV-1_NL4-3 _infection studies in a second T-cell line, C8166 (Fig. 2A, Table 1).

**Table 1:** Selectivity indices of aqueous Lamiaceae extracts in different HIV-1 infection models

**Extract**	**Cells**	**IC_50 _(%)^*a*^**	**CC_50 _(%)^*b*^**	**Selectivity Index^*c*^**
**Lemon balm**	SupT1	0.020 ± 0.012	0.38 ± 0.086	103 ± 66 (6)
	C8166	0.033 ± 0.020	> 1	> 63 ± 19 (4)
	C8166^Entry^	0.004 ± 0.002	> 1	> 1834 ± 930 (4)
	HLAC	0.054 ± 0.016	9.433 ± 0.463	210 ± 42 (3)^*d*^
**Peppermint**	SupT1	0.190 ± 0.124	1.700 ± 0.497	71 ± 29 (6)
	C8166	0.026 ± 0.016	> 1	> 191 ± 87 (2)
	C8166^Entry^	0.005 ± 0.001	> 1	> 208 ± 40 (3)
	HLAC	0.666 ± 0.159	> 10	> 19 ± 6 (3)^*d*^
**Sage**	SupT1	0.099 ± 0.066	1.742 ± 0.526	73 ± 28 (4)
	C8166	0.016 ± 0.005	> 1	> 82 ± 20 (4)
	C8166^Entry^	0.009 ± 0.004	> 1	> 198 ± 70 (3)
	HLAC	1.157 ± 0.672	> 10	> 22 ± 9 (3)^*d*^

^*a*^Inhibitory concentration 50, derived from experiments performed as shown in Figs. 1, 2A, 2B, 3A, 4B.

^*b*^Cytotoxic concentration 50, derived from experiments performed as shown in Figs. 1, 2A, 2B, 4C.

^*c*^Selectivity indices (SI) determined by dividing the CC_50 _by the corresponding IC_50 _value from each individual experiment (and not the arithmetic means of CC_50 _and IC_50 _values shown in this table). IC_50_, CC_50 _and SI values shown represent the arithmetic mean SEM of (n) experiments.

^*d*^Shown is the SI for 3 out of 4 independent experiments. CC_50 _values in the 4^th ^experiment were: Lemon balm CC_50 _> 1, sage and peppermint CC_50 _> 3.

We then extended our analyses to the primary human lymphoid aggregate culture (HLAC) model. These dispersed tonsil tissues are permissive for HIV-1 infection independent of exogenous stimulation [10,11] providing a biologically relevant experimental *ex vivo *model system of HIV infection with preserved endogenous cytokine milieu and a heterogeneous pool of primary target cells. All three extracts markedly inhibited HIV-1 infection in HLAC (Fig. 2B, Table 1). Interestingly, Lamiaceae extracts displayed no cytotoxicity in HLAC up to concentrations of 10% in the majority of experiments performed (Fig. 2B, Table 1).

Furthermore, a continuous treatment of monocyte-derived macrophages with lemon balm extract for 16 days at concentrations up to 3% was not cytotoxic (Fig. 2E). More importantly, infection of these primary cells by the macrophage-tropic R5 HIV-1 strain YU-2 was drastically inhibited in the presence of the extract with an endpoint IC_50 _of 0.018% (Fig. 2C, D). Collectively, these experiments identify a potent antiviral activity of aqueous leaf extracts from three well known species of the Lamiaceae family against X4 and R5 HIV-1 strains at non-cytotoxic concentrations in *in vitro *and *ex vivo *primary cell models of HIV-1 infection.

### Aqueous Lamiaceae extracts target the HIV-1 virion

The HIV-1 life cycle is characterized by an ordered sequence of events that offers multiple theoretical opportunities for antiviral agents to interfere with replication. To gain first insight into the mode of action of the antiviral activity of Lamiaceae extracts, we performed a side-by-side comparison of, on one hand, the effect of extract exposure of both virions and target cells, in principle as described above (Fig. 1), with, on the other hand, a second experimental set-up, in which target cells were first exposed to extracts for 1 h and extracts then washed off prior to HIV-1 challenge.

**Figure 3:**
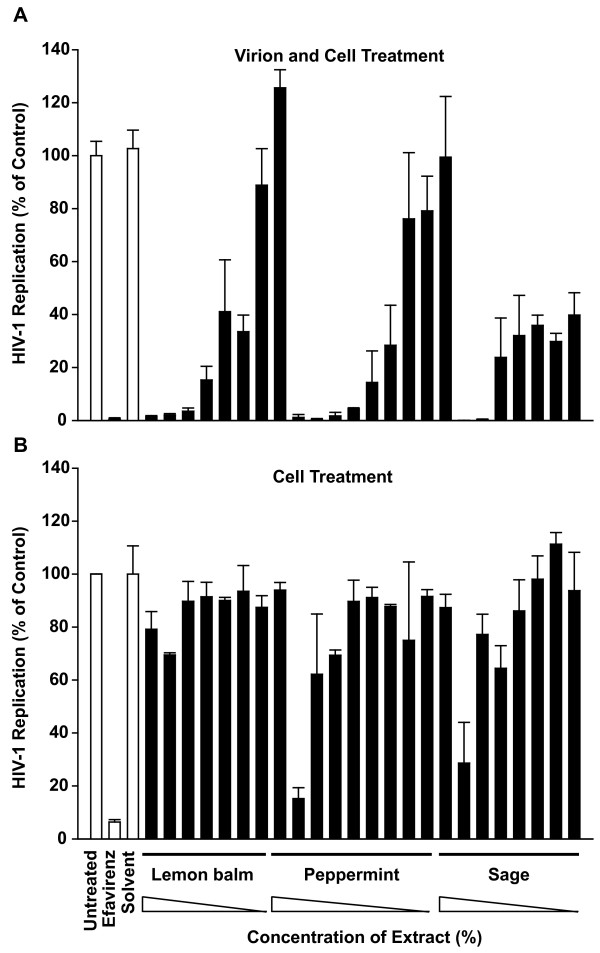
**Extract treatment of HIV-1 particles and target cells, but not of target cells alone, inhibits HIV-1 replication**. (A) Particle and cell treatment. HIV-1_NL4-3 _stocks were exposed to aqueous extracts at concentrations from 0.001 to 0.3% (lemon balm), 0.006 to 3% (peppermint), or 0.006 to 1% (sage), solvent only, or efavirenz for 1 h, and then added to Sup-T1 T-cells. (B) Cell treatment. Alternatively, Sup-T1 cells were directly exposed to aqueous extracts, solvent, or overnight. efavirenz for 1 h. Subsequently, cells were washed and challenged with HIV-1_NL4-3 _Analysis of HIV-1 replication was performed as described in the legend to Fig. 1. Shown are arithmetic means ± SD relative to untreated controls (set to 100%) from one experiment. The inscription for the x-axis of (B) applies also to the x-axis of (A).

As seen before, extract exposure of both virions and target T-cells inhibited HIV-1 replication in a concentration-dependent manner down to background levels (Fig. 3A). This degree of inhibition was also observed in control cultures treated with the reverse transcriptase inhibitor efavirenz at a high concentration (10 μM). In contrast, mere treatment of target cells with aqueous extracts prior to infection had no (lemon balm; Fig. 3B) or a drastically reduced antiviral effect (56-fold for peppermint, >250-fold for sage; Fig. 3B) compared to the treatment of virions and cells (Fig. 3A), suggesting that an anti-HIV activity on target cells is either absent or only transient in nature. As a standard control, a MTT viability assay performed in parallel gave no indication of cytotoxicity at the depicted extract concentrations (data not shown). These experiments suggested that direct exposure of HIV-1 to the extracts rather than an extract-mediated alteration of the target cell or an effect on a later step in the viral replication cycle may be a prerequisite for their antiviral activity.

### Virion fusion is inhibited by aqueous Lamiaceae extracts

In a next step, we employed a sensitive HIV virion-fusion assay to assess whether the earliest events in the infection process, i.e. the interaction of virions with the HIV receptor complex and subsequent membrane fusion, are affected by extract exposure. This assay system is based on the incorporation of β-lactamase-Vpr chimeric fusion proteins (BlaM-Vpr) into replication-competent HIV-1 virions during virus production and their subsequent delivery into the cytoplasm of the target cells as a consequence of virion fusion. BlaM-Vpr-mediated cleavage of the fluorescent CCF2 substrate, which is loaded into target cells, allows a sensitive detection of virus entry and can be quantified by flow cytometry [12].

**Figure 4:**
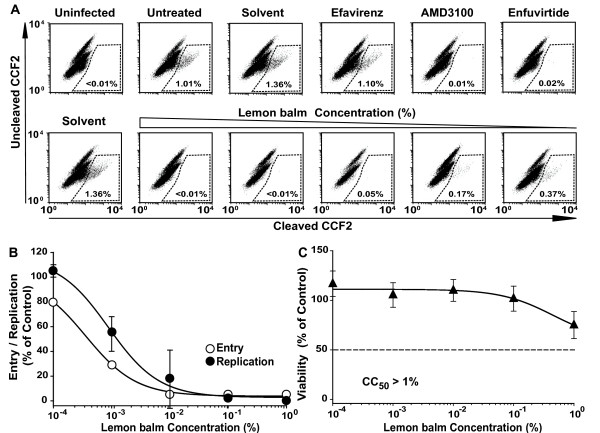
**The aqueous lemon balm extract efficiently inhibits HIV-1 virion-fusion and results in a concordant inhibition of HIV-1 replication**. (A) HIV-1_NL4-3 _virions carrying BlaM-Vpr were exposed to lemon balm extract at concentrations from 0.001 to 1% for 1 h at 37°C, and subsequently added to C8166 T-cells. Alternatively, T-cells were pretreated with AMD3100, enfuvirtide, or efavirenz for 15 min. Virion fusion was analyzed by multiparameter flow cytometry as reported [17, 29]. Shown are representative FACS dot plots for the detection of CCF2 substrate cleavage. (B) A replication assay with an experimental set-up analogous to that described in the legend to Fig. 1 was performed with the identical virus stock used in the virion-fusion assay. (C) MTT viability assay. Each experiment was performed in triplicate, and four independent experiments were conducted. Given are arithmetic means ± SD from one experiment.

**Figure 5:**
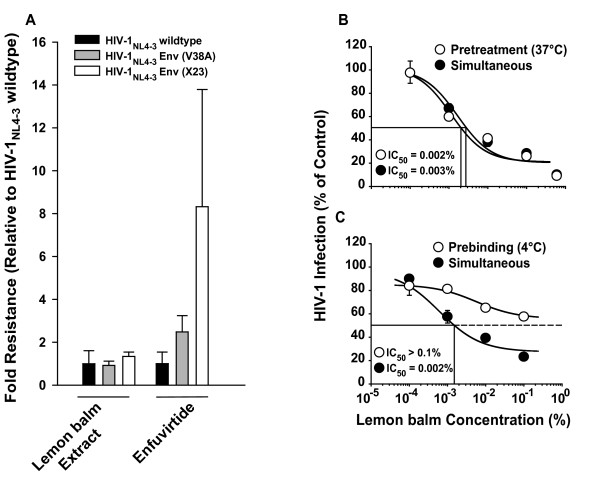
**The aqueous lemon balm extract is fully active against HIV-1 mutants with reduced enfuvirtide sensitivity, but looses potency against surface-bound virions**. HIV-1_NL4-3 _wildtype, HIV-1_NL4-3 _Env (V38A) and HIV-1_NL4-3 _Env (X23) virions were exposed to lemon balm extract at concentrations from 0.00001 to 1% for 1 h at 37°C and subsequently added to TZM-bl cells. Alternatively, TZM-bl cells were pretreated with enfuvirtide at concentrations ranging from 0.0032 to 10 μM for 15 min and then inoculated with HIV-1_NL4-3 _wildtype and mutants. TZM-bl cells were washed the following day. 48 h post infection cells were lysed and the luciferase activity was quantified. Each experiment was performed in triplicate, and four independent experiments were conducted. IC_50 _values for HIV-1_NL-43 _wildtype infections were arbitrarily set to 1 and a factor of difference was calculated for the mutant strains, corresponding to the degree of resistance, for each experiment. Shown are arithmetic means ± standard error of the mean (SEM) of the degree of resistance from four independent experiments. (B) HIV-1 GFP reporter viruses pseudotyped with JR-FL Env were either pretreated with lemon balm extract for 1 h at 37°C and then added to TZM-bl cells or added to target cells simultaneously with the extract. (C) Alternatively, virions were either prebound to cells for 2 h at 4°C, washed, exposed to extract and then shifted to 37°C, or added to cells simultaneously with the extract at 37°C. (B, C) The experiments shown are representative for 2–4 independent experiments and the arithmetic mean ± SD (n = 3) is given.

Here, the validity and specificity of the assay was confirmed employing several controls, including the CXCR4 coreceptor antagonist AMD3100, the fusion inhibitor enfuvirtide, and efavirenz (Fig. 4A, top panels). Enfuvirtide is a synthetic peptide in clinical practice corresponding to a region in the transmembrane subunit of the HIV-1 envelope glycoprotein [13] (see also Fig. 5). Exposure to extracts from sage, peppermint (Table 1 and data not shown), and lemon balm (Fig. 4A, B, Table 1) induced a very potent inhibition of HIV-1 fusion in C8166 T-cells in the absence of cytotoxicity (Fig. 4C, Table 1). The SIs ranged from >198 to >1834 (Table 1). Notably, the antiviral effect of aqueous Lamiaceae extracts was independent of the presence of fetal calf serum since its omission during both the extract-virion incubation and during the challenge period of target cells had no impact on the ability of extracts to inhibit HIV-1 fusion (data not shown).

To address whether the pronounced effect on HIV-1 entry could fully account for the antiviral effect seen on HIV-1 replication, a HIV-1_NL4-3 _BlaM-Vpr stock was exposed to different concentrations of lemon balm extract and, subsequently, analyzed separately in the virion-fusion assay and in the HIV-1 replication assay on C8166 T-cells. The dose-response curves of both analyses were remarkably similar (Fig. 4B). This suggested that the inhibition of HIV-1 entry is the major mechanism by which antiviral activity is achieved by lemon balm extract.

### Lemon balm extract efficiently inhibits enfuvirtide-insensitive HIV-1 strains

Based on the finding that Lamiaceae extracts affect viral infectivity by inhibiting HIV-1 fusion, we tested whether their virucidal activity may be different for HIV-1 strains carrying *env *mutations which confer partial resistance to the fusion inhibitor enfuvirtide [14,15]. Both HIV-1_NL4-3 _*env *mutant strains (V38A, X23) were as susceptible to the antiviral effect of lemon balm extract as the wildtype strain in a luciferase reporter assay on TZM-bl cells (Fig. 5A), while mutants required higher concentrations of enfuvirtide to inhibit infection compared to the wildtype strain, as reported previously [14,15].

### The anti-HIV-1 activity in lemon balm extract is rapid, but reduced against surface-bound virions

To study the kinetics of the extract-mediated antiviral activity, HIV-1 virions were either pretreated with lemon balm extract for 1 h at 37°C prior to addition to target cells, reflecting the standard experimental set-up described for the above experiments, or added to target cells simultaneously with the extract. Remarkably, both conditions displayed an equivalent antiviral potency (Fig. 5B), suggesting an immediate impact of the activity. In contrast, surface-bound virions, which had been preadsorbed to TZM-bl cells at 4°C, were at least 100-fold less susceptible to the extract-mediated inactivation (Fig. 5C). Collectively, free virus particles are the prime target of a rapidly acting antiviral activity present in Lamiaceae extracts.

### Lemon balm extract does not inhibit HIV-1 Env-mediated cell-cell fusion

**Figure 6:**
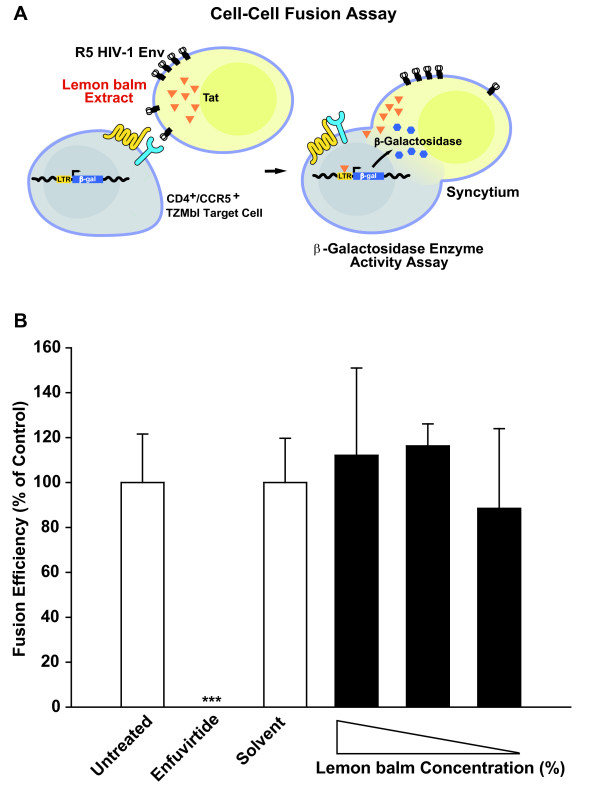
**Lemon balm extract does not inhibit HIV-1 Env-mediated cell-cell fusion**. (A) Schematic of assay used for the quantification of Env-mediated cell-cell fusion. The fusion efficiencies of TZM-bl target cells, expressing the HIV receptor complex, cocultured with CHO Tat cells, transiently expressing the JR-FL Env proteins, were determined via β-galactosidase enzyme activity in relation to the protein concentration. (B) Following overnight transfection, CHO Tat cells expressing JF-FL Env were treated either with different concentrations of lemon balm extract (0.6% (highest non-toxic concentration), 0.1%, or 0.06%), solvent, or enfuvirtide (positive control) for 1 h prior to addition of TZM-bl cells and during the coculture period. The relative fusion efficiency of untreated cells was set to 100% and the arithmetic mean ± SD (n = 4) are given. The experiment shown is representative for three independent experiments. *** Student's *t*-test; p < 0.001.

Next, we explored whether the antiviral effect on virion fusion could also be recapitulated in a classical cell-cell fusion assay, in which CHO donor cells express the JR-FL Env as well as the viral transactivator Tat, and fusion to CD4/CCR5-expressing TZM-bl target cells results in a Tat-mediated expression of the *β*-galactosidase reporter, the relative enzymatic activity of which can be quantified (Fig. 6A) [16,17]. Donor cells were first pretreated with different concentrations of lemon balm extract and then receptor-bearing target cells were added. The mixed cell population was cultivated in the continuous presence of extract for 24 h until analysis. Providing a positive control, enfuvirtide potently inhibited the Env-mediated fusion of donor and target cells (Fig. 6B). In contrast, lemon balm extract even at the highest non-toxic concentration did not interfere with fusion activity. Thus, the extract-mediated antiviral activity does not appear to affect donor cell membranes in general, but may have some specificity for free virions.

### Aqueous extracts inhibit HIV-1 particles carrying R5 Envs or the glycoprotein from vesicular stomatitis virus, as well as Moloney murine leukemia virus

**Figure 7:**
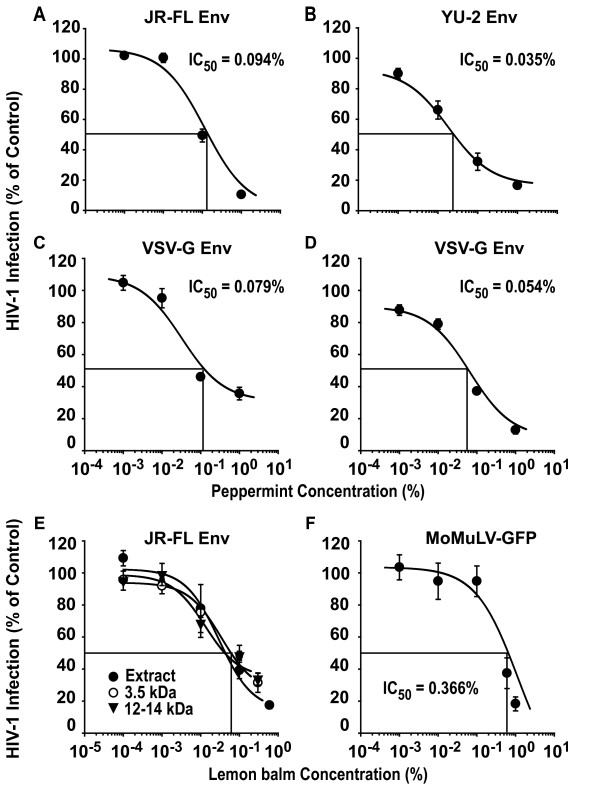
**The antiviral activity of aqueous Lamiaceae extracts extends to different R5 HIV-1 Envs VSV-G pseudotypes, and MoMLV**. HIV-1 GFP reporter viruses, pseudotyped with either R5 HIV-1 Envs from (A) JR-FL, (B) YU-2, or (C, D) VSV-G, were exposed to peppermint extract at the indicated concentrations for 1 h at 37°C and subsequently added to TZM-bl cells. Cells were washed the following day and analyzed for GFP expression by flow cytometry on day 3 post infection. (E) Lemon balm extract was dialyzed overnight against water at molecular weight cut-offs of 3.5 kDa or 12 kDa and then tested for antiviral activity as described above. (F) Replication-competent ecotropic MoMLV-GFP was exposed to lemon balm extract prior to addition to primary rat T-cells. The level of infection of cultures was scored on day 4 post infection and the percentage of GFP-positive cells of solvent-treated controls was set to 100%. Experiments were performed in triplicate, and 2–3 independent experiments were conducted. Given are arithmetic means ± SD from one representative experiment (two independent experiments are shown for VSV-G).

Having mapped a major antiviral activity of aqueous extracts to the unbound virus particle, we explored whether the infection of HIV-1_NL4-3 _virions pseudotyped with Envs from two CCR5-using strains or the heterologous VSV-G were affected by aqueous extracts in single-round infection assays. The peppermint extract inhibited the infection of TZM-bl cells by HIV-1 GFP reporter viruses carrying the R5 Envs from either JR-FL (Fig. 7A; IC_50 _= 0.184 ± 0.037% (n = 3)) or YU-2 (Fig. 7B; IC_50 _= 0.028 ± 0.003% (n = 3)) in a concentration-dependent manner. Of mechanistic importance, the infection by VSV-G pseudotypes was also potently inhibited (Fig. 7C, D; IC_50 _= 0.079 ± 0.028% (n = 3)). Furthermore, the infection of an enveloped gamma-retrovirus, ecotropic Moloney murine leukemia virus encoding GFP (MoMLV-GFP) [18], was also inhibited by an aqueous Lamiaceae extract (Fig. 7F). In contrast, the infection by a non-enveloped adenovirus type 5 reporter virus was not or only slightly impaired, and an IC_50 _value could not determined in 3 out of 4 independent experiments (data not shown). In summary, Lamiaceae extracts inhibit virions with diverse envelopes (HSV [8], HIV, VSV-G, MoMLV), but are considerably less potent against a non-enveloped adenovirus. Collectively, this suggests the virion membrane as the prime antiviral target of these plant extracts.

Since it is not known which component(s) mediate(s) the anti-HIV activity within the three Lamiaceae extracts, we wondered whether they could act synergistically. To this end, HIV-1 GFP reporter viruses were exposed to concentrations of 0.1% or 0.6% of either extracts from sage, lemon balm or peppermint, or a mix of all three extracts at the identical final concentration, and assayed on TZM-bl cells. Within each of these two concentration groups, the degree of inhibition by all four extract conditions was statistically indistinguishable (data not shown), thus providing no evidence for a synergism in the extracts' anti-HIV-1 activity. As an additional characterization, extract dialysis suggested that the active antiviral compounds in lemon balm extract are larger than 12 kDa (Fig. 7E).

### Virion stability and virion-associated levels of Env and processed Gag are not affected by lemon balm extract

**Figure 8:**
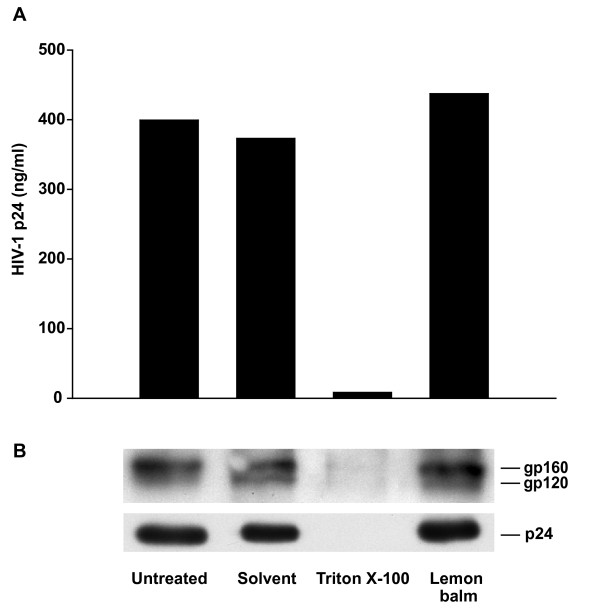
**Extract treatment does not affect particle stability, content of Env and processed Gag**. Purified HIV-1_NL4-3 _virions were exposed either to aqueous lemon balm extract (1%), solvent (1%), or Triton X-100 (0.5%) for 1 h at 37°C and subsequently ultracentrifuged through a 20% sucrose cushion. Pellets were resuspended in PBS and analyzed either by (A) anti-p24 ELISA or (B) subjected to Western blot analysis.

We then investigated whether aqueous Lamiaceae extracts may alter the integrity of HIV-1 particles that could result in an increased fragility of virions. To test this, HIV-1_NL4-3 _virions were exposed to lemon balm extract, solvent or, as an informative control, the detergent Triton X-100, for 1 h and subsequently centrifuged through a 20% sucrose cushion. Neither exposure to lemon balm extract nor solvent significantly altered the amount of virions that could be recovered in the pellet fraction relative to the untreated control (Fig. 8A), while, expectedly, exposure to Triton X-100 drastically diminished the levels of pellet-associated p24. Moreover, immunoblotting analyses of pellet fractions with anti-Env and anti-Gag antibodies provided no evidence for extract-induced changes in the relative levels of virion-associated gp160 and gp120, or levels of p24 (Fig. 8B), with no detectable Pr55^Gag ^signal under all treatment conditions (not shown). Thus, lemon balm extract does not appear to grossly affect the stability of virions, Gag processing, or Env shedding.

### Virion density is enhanced by aqueous Lamiaceae extracts

**Figure 9:**
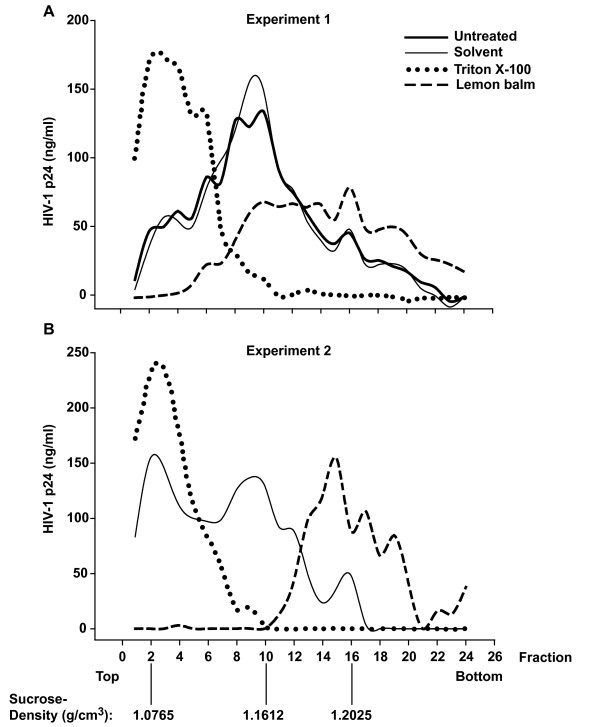
**Extract treatment enhances the density of HIV-1 particles**. Purified HIV-1_NL4-3 _virions were exposed either to aqueous lemon balm extract (1%), solvent (1%), or Triton X-100 (0.5%) for 1 h at 37°C and subsequently ultracentrifuged in a sucrose-density equilibrium gradient. Gradient fractions were collected from top to bottom and fractions analyzed by anti-p24 ELISA. Results from two independent experiments are shown. The inscription for the x-axis of (B, experiment 2) applies also to the x-axis of (A, experiment 1).

To allow the detection of more subtle extract-induced changes on the density of HIV-1 particles, we performed continuous sucrose-density equilibrium gradient analyses. As a reference, untreated HIV-1 virions accumulated at a sucrose density of ~1.16 g/cm^3 ^(Fig. 9), consistent with previous studies which reported densities ranging from 1.15 to 1.18 g/cm^3 ^for intact HIV-1 virions [19,20]. Moreover, Triton X-100 resulted in a destruction of virions resulting in a p24 accumulation at the top of the gradient (~1.08 g/cm^3^). Relative to the solvent control, exposure to the aqueous extract from lemon balm induced a drastic shift in the HIV-1 gradient profile with an accumulation of virions at a markedly higher sucrose density at equilibrium (~1.20 g/cm^3^, Fig. 9, two independent experiments shown). Similar findings were obtained for peppermint extract (data not shown). Collectively, an enhanced density of HIV-1 virions without an apparent impact on their integrity or Env content is the key physicobiochemical change induced by extract exposure.

## Discussion

This study identifies a potent anti-HIV-1 activity in aqueous extracts from dried leaves of three well known plants. The major antiviral activity of these tea-like extracts from lemon balm, peppermint, and sage targets the HIV-1 virion. The extracts inhibited the capacity of virions to enter into target cells at concentrations typically two orders of magnitude below the cytotoxic concentrations. A strong antiviral activity was observed for different types of virions carrying a broad spectrum of viral envelopes, but not against the non-enveloped adenovirus type 5.

The cardinal antiviral activity of aqueous Lamiaceae extracts appears to be virucidal and through this mechanism affecting the interaction of the virion with the cell. The activity is rapid since even simultaneous addition of virus and extract to cells allowed for full antiviral potency. In contrast, surface-bound virions were largely protected, suggesting that following engagement of the HIV receptor complex, the extract-induced alterations of the virion can either no longer be exerted or loose their functional consequence. At this point, reversible or transient effects of the extracts on the cell surface, that may additionally impede virion fusion, or on intracellular steps of the HIV-1 replication cycle cannot be entirely excluded. Since these extracts inhibited HIV-1 virions carrying different X4 and R5 HIV-1 Envs as well as the heterologous VSV-G, interfered with MoMLV infection, and were previously shown to be highly active against HSV-1 and HSV-2 [8], a mode of action that does not specifically target the viral envelope seems likely. Of particular note, while the extracts inhibited fusion of HIV-1 particles with cells, we found that lemon balm extract was completely ineffective in blocking HIV-1 Env-mediated cell-cell fusion.

This indicates that the context of the virion membrane may be a prerequisite or even the prime target for the antiviral activity exerted by the Lamiaceae extracts.

Mechanistically, extract exposure induced an accumulation of virions at a higher sucrose density. Physicobiochemically, this cannot be due to an aggregation of particles in a gradient at equilibrium. Instead, this finding suggests an enhancement of the density of HIV-1 particles and this appears to be the most likely correlate of the reduced infectivity, although a causal link cannot be established at this point. Such a density enhancement could, for example, be due to chemical modifications of the proteins, glycans [21], or lipids of virions by specific extract components, or due to the attachment of extract components to the virus particle. Interesting in this context, Campbell and colleagues showed that the experimental modification of the lipid composition of HIV-1 virions, i.e. by methyl-β-cyclodextrin-induced cholesterol depletion and/or exogenous lipid replenishment, could result in a higher virion density and a reduction in infectivity [22,23]. Notably, the enhancement in virion density relative to untreated controls appeared to be at least as pronounced following treatment with lemon balm extract (Fig. 9) as the published effects on virions with exogenously modified lipids [22,23]. Collectively, a plausible mechanistic model is that the extract-induced increase in the density of enveloped virions prevents their attachment to cells.

At antiviral concentrations that were highly effective in *in vitro *and *ex vivo *model systems of HIV-1 infection these inexpensive aqueous extracts from leaves of abundant species of the Lamiaceae family are colorless and odorless, fulfilling basic criteria for the development of a topical microbicide. In addition, Lamiaceae extracts as natural products are widely used in food products, including herbal teas and ice creams, as well as in cold medicine and mild sedative agents, indicating a low systemic toxicity and established acceptance in the general population. A number of candidates, including membrane-disrupting detergents and acidifying agents, which are currently under development as topical microbicides, inactivate HIV particles directly [2]. As an interesting comparison, several peptides with anti-HIV activity have been reported that directly interact with the transmembrane subunit of the envelope glycoprotein, preventing virion fusion. Such peptides include SJ-2176 and T20 (enfuvirtide) [24,25] derived either from the C-terminal heptad repeat of gp41, or, VIRIP, a naturally occurring peptide identified in human hemofiltrate, which also targets gp41 [26]. While both Lamiaceae extracts and these antiviral peptides prevent fusion of HIV particles, their mode of action is quite distinct. The gp41-targeting peptides, on one hand, are highly selective for HIV-1, display virtually no cytotoxicity, and still allow virus binding to the receptor. The complex aqueous extracts, on the other hand, have a broader antiviral activity that is not selective for a specific viral envelope, display moderate cytotoxicity, and appear to modify the particle without affecting its integrity such that already virus attachment may be inhibited.

The SI values of all three Lamiaceae extracts are encouraging for a complex plant extract ranging from >19 to >1834. For comparison, SI values of >40, >50, and >333 have been reported for the topical HIV microbicide candidates cellulose sulphate, polymethylenehydroquinone sulfonate, or the mandelic acid condensation polymer SAMMA for the infection of primary target cells by HIV-1_Ba-L _[3]. Interestingly, SI values for our aqueous extracts from sage, lemon balm and peppermint ranged from 65 to 2037 for HSV-2 infection [8]. Other investigators have made important contributions to the design and advancement of topical microbicides that can target both HIV and HSV-2 [3], with the most recent addition of so called molecular umbrellas, including Spm8CHAS [27].

It will be interesting to explore by which molecular mechanism these extracts enhance the density of HIV-1 particles. Based on the dialysis studies the active components appeared to be >12 kDa and attempts to identify individual compounds with high antiviral activity by bioguided fractionation of aqueous Lamiaceae extracts may enhance their utility as a lead for the development of a topical microbicide for the prevention of transmission of two major sexually-transmitted pathogens, HSV-2 and HIV-1.

## Methods

### Viral stocks

The molecular HIV-1 clone of pNL4-3 and its derivative pNL4-3 E^- ^GFP, the latter carrying an *egfp *gene within the *nef *locus driven by the 5'-LTR, were obtained from Dr. Malcom Martin (National Institutes of Health (NIH), Bethesda, MD) and Dr. Nathaniel Landau (New York University, New York, NY) respectively, via the NIH AIDS Research and Reference Reagent Program. Pseudotyping with VSV-G, JR-FL Env and YU-2 Env was performed as reported [28]. The molecular clones of HIV-1_NL4-3 _Env (V38A) and HIV-1_NL4-3 _Env (X23) were a kind gift from Dr. Matthias Dittmar (University of Heidelberg, Heidelberg, Germany) [15]. The X23 *env *gene was derived from a T20-naive HIV-positive individual [14,15]. Infectious stocks from proviral DNA were generated by transfection of proviral HIV plasmids into 293T cells as described [28]. HIV-1_NL4-3 _virions containing β-lactamase-Vpr chimeric fusion proteins (BlaM-Vpr) were produced by triple-transfection of 293T cells with pNL4-3 proviral DNA (60 μg), pBlaM-Vpr (20 μg), and pAdVantage (8 μg) vectors (Promega, Madison, WI) per 15-cm^2 ^dish by calcium phosphate DNA precipitation as described [17,29]. Two days after transfection, culture supernatants were harvested and viral stocks were concentrated using Centricon^® ^Plus-70 spin columns (Millipore, Billerica, MA). After concentration, HIV-1 virions were purified through a 20% or 30% sucrose cushion (44.000 *g*, 4°C, 60 min), and the virion-enriched pellet was resuspended in phosphate-buffered saline (PBS) and stored at -80°C. The p24 concentration of HIV-1 stocks was determined by antigen enzyme-linked immunosorbent assay (ELISA) as reported [30]. The construction of the replication-competent MoMLV-GFP reporter virus has been reported [18].

### Cell lines, monocyte-derived macrophages and primary rat T-cells

All cell lines and primary macrophages were cultivated under standard conditions in Dulbecco's modified Eagle medium (293T, TZM-bl, MDM) or RPMI 1640 (Sup-T1, C8166, CHO Tat) (both media from GIBCO, Karlsruhe, Germany) supplemented with 10% fetal bovine serum (Invitrogen, Karlsruhe, Germany), 1% penicillin-streptomycin, and 1% L-glutamine (both from GIBCO). Cultures of monocyte-derived macrophages were prepared from Ficoll gradient-purified peripheral blood mononuclear cells isolated from individual, healthy HIV-, HCV-, HBV-seronegative blood donors (DRK Blutspendezentrale, Mannheim, Germany) by adherence and were differentiated in the presence of 10% human AB serum (Invitrogen) for 6–8 days as reported [28,31]. Cultures of primary rat T-cells were generated as reported [28,31].

### Human Lymphoid Aggregate Culture(HLAC) from tonsil

Tonsil tissue was removed during routine tonsillectomy from HIV-, HBV-, HCV-negative patients with informed consent. To prepare HLAC, tonsil tissue was mechanically dispersed by cutting tissue in 2- to 3-mm blocks and passing them through 40-μm cell strainers (BD Falcon, Belgium). Cells were washed in PBS, and 2 × 10^6 ^cells were plated in 96-well V-bottom plates (Corning Incorporated, New York, NY) in a final volume of 200 μl. Culture medium (RPMI 1640 containing 15% fetal bovine serum, 1% L-glutamine, 1% fungizone, 1% gentamycin (all from GIBCO), 0.25% ampicillin (Roth, Karlsruhe, Germany), 1% non-essential amino acids, and 1% sodium pyruvat (both from Invitrogen)). Detailed cultivation methods have been reported [10,11]. One day after tonsil preparation, the HLAC was inoculated with HIV-1 (5 ng p24 per 2 × 10^6 ^cells per well). Where indicated, HIV stocks were preincubated with aqueous Lamiaceae extracts or solvent alone for 1 h at 37°C prior to infection. Following overnight infection cells were washed and the culture medium was subsequently changed every two days without dispersing the pellet. At the same time intervals supernatant samples were harvested and stored at -20°C for subsequent analysis by p24 ELISA.

### Aqueous extracts from species of Lamiaceae

Dried leaves from lemon balm (*Melissa officinalis *L.), peppermint (*Mentha *× *piperita *L.), and sage (*Salvia officinalis *L.) were purchased from Caesar & Lorenz (Hilden, Germany). All plants were identified by microscopy and chromatography according to their monographs in the Pharmakopoea Europoea. Aqueous extracts were prepared as described previously [8]. Briefly, boiling water (100 ml) was added to dried leaves (10 g) and incubated for 15 min, subsequently filtered and cooled down. The resulting extracts were sterile filtered, aliquoted, and stored at -20°C.

### Antiviral drugs

Enfuvirtide (Fuzeon^®^, (T20) Roche, Indianapolis, IN) was freshly dissolved in H_2_O at 9 mg/ml. Efavirenz (Sustiva^®^, Bristol-Myers Squibb, Jacksonville, FL) was purchased as a drinking solution at 30 mg/ml and diluted in culture medium. AMD3100 was a kind gift from Dr. José Esté (Badalona, Spain).

### Treatment of virus particles and cells

First, virus stocks were incubated with the indicated concentrations (v/v) of aqueous Lamiaceae extracts or H_2_0 as solvent control for 1 h at 37°C. Subsequently, this suspension was mixed with an equivalent volume of culture medium (1:1) and added to target cells (5 ng p24 per 4 × 10^4 ^cells). Following overnight exposure, cells were washed and cultivated for 4 more days. Productive HIV-1 infection was assessed by the p24 concentration in culture supernatants.

### Exclusive treatment of cells

Cells were exposed to the indicated concentrations of aqueous extracts in culture medium for 1 h at 37°C. Subsequently, the supernatant was discarded, cells were washed once with PBS and challenged overnight with HIV-1 (5 ng p24 per 4 × 10^4 ^cells).

### Cell viability assay

In parallel to all infection assays, uninfected cells were cultivated in the presence of aqueous Lamiaceae extracts at the identical concentrations used in the infection assays, or in the presence of solvent alone (reference control). Following overnight exposure, cells were washed and cultivated for 3 more days, at which time the cytotoxicity was determined by quantifying the amount of a formazan product metabolized by viable cells from the 3-(4,5-dimethylthiazol-2-yl)-2,5-diphenyltetrazolium bromide (MTT) solution (Sigma) as reported [9].

### HIV-1 virion-fusion assay

The flow cytometry-based HIV-1 virion-fusion assay was performed essentially as described [12,17,29]. Briefly, C8166 T-cells were pretreated with enfuvirtide (2 μM), AMD3100 (10 μM), or efavirenz (10 μM) for 15 min. Alternatively, HIV-1_NL4-3 _BlaM-Vpr virions were pretreated for 1 h at 37°C with the indicated concentrations of aqueous lemon balm extract or solvent. Subsequently, C8166 T-cells were exposed to HIV-1_NL4-3 _BlaM-Vpr virions (40 ng p24 per 2 × 10^6 ^cells) for 6 h, washed and then loaded with CCF2/AM dye overnight. Fusion was monitored with a three-laser BD FACSAria Cell Sorting System (Becton Dickinson, San Jose, CA).

### Luciferase reporter virus assay

HIV-1_NL4-3 _wt virions, or T20-insensitive HIV-1_NL4-3 _Env (V38A) or HIV-1_NL4-3 _Env (X23) virions were exposed to lemon balm extract at concentrations from 0.00001 to 1% or solvent for 1 h at 37°C and subsequently added to TZM-bl cells, carrying an LTR-driven *firefly luciferase *gene. In parallel, TZM-bl cells were pretreated with enfuvirtide at concentrations from 0.0032 to 10 μM for 15 min and then challenged with virus. TZM-bl cells were washed the following day. 48 h after infection a luciferase reporter assay system (Promega) was used to monitor the enzymatic activity.

### Cell-Cell fusion assay

This assay was performed in principle as described [16,17]. Briefly, CHO Tat cells were transiently transfected with expression plasmids encoding for HIV-1 JRFL Env or HIV-1 YU-2 Env together with pCMV-Rev. One day later, transfected CHO Tat cells and TZM-bl cells, the latter stably expressing CD4, CCR5, and an LTR-driven *β-galactosidase *gene, were harvested and cocultured in a 1:1 ratio in 96-well plates (2 × 10^4 ^cells per well in 200 μl of a 1:1 mixture of complete RPMI and DMEM culture medium). Where indicated, transfected CHO Tat cells were exposed to aqueous lemon balm extract or solvent for 1 h at 37°C prior to mixing. The following day, cells were washed once with PBS, and the β-galactosidase enzyme activity and protein concentration in cell lysates were determined with the Galacto-Star™ System (Applied Biosystems) and the BCA™ Protein Assay Kit (Pierce), respectively. The luminometric activity was analyzed with a Luminoskan Ascent (Thermo Labsystems) luminometer and Ascent Software 2.0.

### HIV-1 single-round infections

TZM-bl cells were seeded at a density of 1 × 10^5 ^cells per well and challenged with single-round HIV-1_NL4-3 _E^- ^GFP reporter viruses (HIV-1 GFP) pseudotyped with JR-FL Env, YU-2 Env, or VSV-G (all 20 ng p24 per well). Prior to infection, virions were exposed to peppermint extract at concentrations ranging from 0.001 to 1% or solvent for 1 h at 37°C. TZM-bl cells were washed the following day. Three days after infection, the percentage of GFP-positive cells was determined on a FACSCalibur using BD CellQuest Pro 4.0.2 Software (BD Pharmingen).

For the dialysis experiment, lemon balm extract was dialyzed for 24 h at room temperature against water at molecular weight cut-offs of 3.5 or 12 kDa (Spectra/Por^® ^Regenerated Cellulose (RC) Dialysis Membranes). Dialysis-induced changes in extract volume were accounted for prior to virus exposure.

Exploring the activity of lemon balm extract on surface-bound viral particles, TZM-bl cells were inoculated with HIV-1 GFP (200 ng p24 per well) and incubated for 2 h at 4°C. Cells were washed and then solvent or lemon balm extract was added at the indicated concentrations and cells were cultivated at 37°C until analysis.

### Western blot analysis

Virions were exposed to lemon balm extract (1%), solvent (H_2_O; 1%), or Triton X-100 (0.5%) for 1 h at 37°C, pelleted through a 20% sucrose cushion by ultracentrifugation (44.000 *g*, 4°C, 60 min) and resuspended in PBS (4°C, 30 min) for subsequent analysis by Western blotting. Briefly, samples were boiled in sodium dodecyl sulphate (SDS) sample buffer, separated by 10% SDS-polyacrylamide gel electrophoresis, and transferred onto a nitrocellulose membrane. After incubation with primary antisera (rabbit anti-gp120 Env, 1:5000 (kind gift from Dr. Valerie Bosch, DKFZ, Heidelberg); rabbit anti-24 Gag, 1:4000 (kind gift from Dr. Hans-Georg Kräusslich)) and secondary antibodies (goat anti-rabbit-horseradish peroxidase, 1:10.000; Dianova, Hamburg, Germany), viral structural proteins were detected with an ECL staining solution as reported [16].

### Sucrose-density equilibrium gradient analysis

HIV-1_NL4-3 _virions (~500 ng p24), which had been purified and enriched by ultracentrifugation through a 30% sucrose cushion, were incubated with either aqueous Lemon balm extract (1%), solvent (H_2_O; 1%), Triton X-100 (0.5%), or left untreated for 1 h at 37°C. Subsequently, the virion suspensions were loaded onto a 20 to 60% linear sucrose gradient (total volume: 3.5 ml). After ultracentrifugation at 44.000 rpm for 16 h at 4°C in a SW60 rotor, 24 fractions of each 150 μl were carefully collected from top to bottom and the p24 concentration was analyzed. The sucrose density of fractions from an additional tube (without virus) run in parallel was determined by refractometry.

## Competing interests

The author(s) declare that they have no competing interests.

## Authors' contributions

SG and OTK designed the study. SG conducted the majority of the experiments. CG performed titration inhibition experiment and provided technical support. SG and SV established and validated the HLAC model system and IB and PP provided tonsillectomy material. SN and JR provided aqueous plant extracts and data on HPLC-UV and LC-MS analyses. JR assisted in interpretation of data. SG and OTK wrote the paper. All authors commented on and approved the final manuscript.
